# Induction of NETosis in ovine colostral PMN upon exposure to *Neospora caninum* tachyzoites

**DOI:** 10.3389/fvets.2023.1176144

**Published:** 2023-06-19

**Authors:** Lukas Demattio, Ivan Conejeros, Daniela Grob, Ulrich Gärtner, Anja Taubert, Carlos Hermosilla, Axel Wehrend

**Affiliations:** ^1^Clinic for Obstetrics, Gynaecology and Andrology of Small and Large Animals, Justus Liebig University Giessen, Giessen, Germany; ^2^Institute of Parasitology, Justus Liebig University Giessen, Giessen, Germany; ^3^Institute of Anatomy and Cell Biology, Justus Liebig University Giessen, Giessen, Germany

**Keywords:** *Neospora caninum*, colostrum, neutrophil extracellular traps, ovine PMN, NETosis

## Abstract

Colostrum is one of the most important factors influencing the health and development of mammalian neonates. It is well-established that leukocytes, including polymorphonuclear neutrophils (PMN), migrate from the mother to the infant via colostrum uptake. In this study, for the first time, we studied the ability of ovine colostral-derived PMN to extrude neutrophil extracellular traps (NETs) against the abortive apicomplexan parasite *Neospora caninum*. Although this cell population plays a significant role in the transmission of maternal innate immunity to neonates, little is known about colostral PMN activities in sheep. However, this cell population is a significant source of the transfer of maternal immunity to the neonate. Colostral PMN continues to exert immunological effects even after transitioning into the colostrum. The present study aimed to investigate the extrusion of NETs by ovine colostral PMN exposed to the apicomplexan parasite, *N. caninum*, which is known to cause devastating reproductive disorders in cattle, small ruminants, wildlife animals, and dogs. The present study is the first to demonstrate that ovine colostral PMN can produce NETs after stimulation with vital *N. caninum* tachyzoites. Ovine colostrum-derived NETs were detected by chromatin staining and antibody-based immunofluorescence staining of NET-specific structures, including neutrophil elastase (NE) and global histones (H1, H2A/H2B, H3, H4), as well as scanning electron microscopy (SEM) analysis.

## 1. Introduction

Colostrum is of vital importance for the health of mammalian neonates (1), not only because of the nutritional properties of this first milk immediately after birth (2) but also due to its role in transferring maternal immunity to neonates (3, 4). This transmission of maternal immunity can be attributed to immunoglobulin uptake via the intestine from the colostrum as well as the uptake of colostral leukocytes from the neonatal intestine (5–7).

In the past, it was believed that colostral leukocytes, mainly polymorphonuclear neutrophils (PMN), entered the colostrum accidentally or via mammary gland inflammation. Currently, it is common knowledge that PMN actively migrates into this mammary secretion during colostrogenesis (8). These alive PMN then migrate after colostrum uptake through the intestinal epithelium into the bloodstream of a newborn (9, 10) and thereafter spread rapidly throughout the entire organism, accumulating particularly in the lymphatic tissue (4, 11, 12). Consequently, it has been determined that colostral PMN passed from the mother to the newborn has a pivotal role in regulating and promoting the maturation of the neonate's immune system (10–14). Neonates that have not absorbed sufficient PMN through colostrum uptake exhibit significantly less effective immune defenses and higher susceptibility to various diseases (12, 14). Despite these facts, little is still known about colostral PMN activities in sheep. Neither the quantity of these cells in ovine colostrum nor their potential effector mechanisms have been determined. To date, the release of neutrophil extracellular traps (NETs) in colostrum-derived PMN has only been studied in canine PMN (4), indicating that this research topic is still in its infancy when compared to NET investigations of mammalian blood PMN (15–17).

NETs are secreted, decondensed nuclear chromatin laced with granular and cytoplasmic proteins that are formed by activated PMN (15, 17, 18). The effect of NETs is based on their structural scaffolding: chromatin contributes to the immobilization of pathogens, while contained antimicrobial proteins can themselves contribute to the deactivation of pathogens (15). NETosis can be triggered by various stimulants, including not only intact pathogens but also parts thereof (19, 20). The first component of the NET cascade is always the activation of the Raf/MEK/ERK system, which leads to the formation of a multimeric NADPH-oxidase (NOX) complex. This simultaneously leads to the formation of reactive oxygen species (ROS). As this process continues, a loss of integrity of the intracellular membranes occurs, releasing granular neutrophil elastase (NE). Afterward, this enzyme has to reach the PMN nucleus to initiate hypercitrullination of histones and subsequent chromatin decondensation. As a result, the decondensed chromatin can no longer be contained by the cell, and it bursts into the extracellular space. The number of NETs formed depends, among other variables, on the stimulus and can be determined using various methods. In addition to fluorescence microscopic or electron microscopic imaging, measuring the free DNA is also a viable option (21).

*Neospora caninum* is a common apicomplexan protozoan that causes abortion in several vertebrate species (22). The main final and definitive host of this apicomplexan parasite is the domestic dog (*Canis familiaris*), but other wild canids (e.g., wolves and coyotes) can also act as final hosts. After gamogony and a massive intracellular release of oocysts into the lumen of the canine small intestine, unsporulated oocysts are excreted through defecation. Numerous herbivorous animals (such as cattle, sheep, goats, New World camelids, and wild cervids) can ingest the exogenous sporulated oocysts when grazing, thus becoming infected as intermediate hosts. In the intermediate host, rapidly replicating tachyzoites are formed, which can cross the placental barrier and infect the fetus. This fetal infection can lead to abortion, especially during the first two trimesters of ovine gestation (23). It is known that blood-derived PMN has lethal effects on another apicomplexan parasite, Toxoplasma gondii, in cattle and sheep (24).

To investigate whether ovine colostrum-derived PMN undergo NETosis after stimulation with various potent NET elicitors, including the abortive *N. caninum* parasite described above, we first isolated PMN from ovine colostrum and then incubated them with the abovementioned elicitors.

## 2. Materials and methods

### 2.1. Parasites

*N. caninum* tachyzoites of the Nc1 strain were cultured in plastic T-25 cm^2^ tissue culture flasks (Greiner) either in either primary bovine umbilical vein endothelial cells (BUVEC) or permanent African green monkey kidney epithelial cells (MARC-145). Living, floating *N. caninum* tachyzoites were collected after 3–5 days of culture from infected host cell layer supernatants, pelleted [400 × g, 12 min at room temperature (RT)], washed three times in sterile PBS, counted in a Neubauer counting chamber (Marienfeld-Superior, Germany), and resuspended in sterile RPMI 1640 medium (Gibco, Berlin) until further use, as previously reported (16). For comparative reasons, heat-inactivated and frozen parasites were also used to stimulate ovine colostral PMN. The parasites were heat-inactivated at 65°C for 10 min and stored at−80°C in 1% DMSO-cell-culture media.

### 2.2. Isolation of ovine colostrum-derived PMN

In total, 30 healthy Merino ewes (*n* = 30) of different parties were included in the present study. All sheep had physiological gestation lengths and gave birth to mature lambs. All udders of the mammary glands were free from the clinical signs of inflammation or tumorous degeneration. The level of colostrum was macroscopically within the normal physiological range. All animals were regular patients of the Clinic for Obstetrics Gynaecology and Andrology of Small and Large Animals of the Justus Liebig University Giessen in Germany. All samples were remnant volumes from routine diagnostics (file number kTV 8-2017).

Colostrum samples were collected as soon as possible after parturition of the first lamb, and colostrum was manually milked from both sides of the udder by stroking the teats with the fingers. The obtained colostrum samples were macroscopically within the normal physiological range. The desired PMN were obtained using density gradient centrifugation, and according to Demattio et al. (4), the exact procedure is shown in the following paragraph: After collection, all samples were filtered using sterile 40 μm nylon filters. The equivalent of the sample volume was supplemented with sterile, cold phosphate buffer saline (PBS). This was followed by centrifugation at 600 × g for 20 min at 4°C. After centrifugation, the fat-containing supernatant was poured off, and the cell pellet was carefully resuspended in cold, sterile PBS. Centrifugation was then repeated twice at 600 × g for 20 min at 4°C. After each centrifugation step. The cells were washed with PBS. The remaining pellet was then resuspended in 1 ml of sterile PBS.

This cell suspension was carefully pipetted onto 250 μl Biocoll^®^ separating solution and centrifuged at 800 × g for 45 min at 20°C. Afterward, the supernatant was decanted, and the PMN and erythrocyte fractions remained at the bottom.

To lyse erythrocytes, the cell pellet was dissolved in 25 ml of sterile, double-distilled water and mixed gently for 40 s to disrupt erythrocytes, as reported in a previous study (4). To restore adequate osmolarity, 4 ml of sterile10 × Hanks balanced solution (HBSS; Biochrom AG, Berlin) was added.

### 2.3. Counting of ovine colostral PMN

To determine the quantity of ovine colostral PMN, cells from 15 healthy Merino sheep ewes (*n* = 15) were manually milked and isolated in the procedure described above. All isolated colostrum-derived PMNs were stained with 250 ml of Turk's solution (Merck, Berlin) and counted in a Neubauer haemocyte counting chamber. The results were obtained in cells per milliliter and compared using a *t*-test.

### 2.4. Scanning electron microscopy analysis

Isolated ovine colostral PMN were co-cultured with vital *N. caninum* tachyzoites for 180 min. Only the cells of one ewe were used for the analysis to avoid cross-reactions of PMN from different animals. For this purpose, a PMN-to-parasite ratio of 1:3 was chosen, according to Villagra-Blanco et al. (16). In each experimental set, 50,000–150,000 cells were used, depending on how many cells could be obtained from the corresponding colostrum sample. The co-culture of cells took place on sterile glass coverslips (10 mm diameter; Thermo Fischer Scientific), which were previously pre-coated with 0.01% poly-_L_-lysine (Sigma-Aldrich). Samples were incubated at 37°C for 180 min under 5% CO_2_ atmosphere conditions. After this incubation, cells were carefully pre-fixed with 2.5% glutaraldehyde (Merck) and thereafter post-fixed with 1% osmium tetroxide solution (Merck). The cells were then cleaned in purified water, dried at a critical point using CO_2_ treatment, and sputtered with gold particles. After these preparatory measures, the cells were imaged using a Philips CL30 scanning electron microscope (SEM). SEM imaging analysis was carried out at the Institute of Anatomy and Cell Biology at Justus Liebig University Giessen, Giessen, Germany. As a positive control for ovine NET induction, one-third of the samples were incubated with calcium ionophore A23187 (Merck) instead of *N. caninum* tachyzoites. As negative controls, cells were isolated, incubated, and processed in the same way but without the addition of any potential stimulant.

### 2.5. *Neospora caninum* tachyzoites-induced colostral NETs visualized using immunofluorescence microscopy analysis

Ovine colostral PMN (*n* = 5, from different animals on different days) were co-cultured with vital *N. caninum* tachyzoites (37°C, 5% CO_2_ atmosphere), ionophores A23187 (Merck), and without any stimulation as the negative control for 180 min. This was conducted on sterile glass coverslips (15 mm in diameter, Thermo Fischer Scientific) pre-coated with 0.01% poly-_L_-lysine (Sigma-Aldrich). After incubation, the cells were fixed in a 1% paraformaldehyde solution (Merck) and stored at 4°C until further processing. DAPI was used to visualize released NET structures in colostral PMN. To visualize NET-specific proteins/histones, immunofluorescence microscopy analysis was performed with specific antibodies against neutrophil elastase (NE) and global histones (i.e., H1, H2A/H2B, H3, H4). For this purpose, the samples were washed three times with sterile PBS solution (Sigma-Aldrich) and then blocked with 2% bovine serum albumin (BSA; Sigma-Aldrich) containing 0.3% Triton-X-100 (Thermo Fischer Scientific) for 60 min at room temperature (RT). The samples were then immersed in a solution containing primary antibodies (PAN-histone, 1:200, Chemico ind. # MAB3422 and NE, 1:200 ABCAM #ab68672) for 20 min at RT. Following this procedure, the samples were washed for another three times with sterile PBS and then incubated with secondary antibodies (Alexa 488 goat anti-mouse IgG #A110011, Alexa 405 goat anti-rabbit IgG #A31556, 1:500, both Invitrogen) for 120 min at RT, which was protected from light. As a positive control, the same procedure was performed on cells incubated with calcium ionophore A23187 (Merck) instead of vital *N. caninum* tachyzoites. As a negative control, PMN was served without stimulation and subjected to the same treatment. After another washing step, the glass coverslips were mounted with Fluoromount-G^®^ (Thermo Fischer Scientific). NET structures were visualized using an inverted IX81 epifluorescence microscope equipped with an XM10 digital camera (both Olympus).

### 2.6. Characterization of different NET phenotypes in colostral PMN

For the characterization and quantification of different NET phenotypes in ovine colostral PMN, such as spread NETs (*spr*NETs), which are in long filaments, diffuse NETs (*diff* NETs), which are over a larger area, and aggregated NETs (*agg*NETs), which are the largest, the protocols previously described by Muñoz-Caro et al. (25) were followed in this study. Ovine colostrum-derived PMNs were stimulated and subsequently analyzed by immunofluorescence microscopy, as described above. For optical quantification, five randomly selected fields of view were examined and evaluated as percentages, following the criteria outlined by Muñoz-Caro et al. (25) and Grob et al. (26).

### 2.7. Phagocytosis test

A commercial phagocytosis test assay (pHrodo^TM^ BioParticles^TM^ Phagocytosis Kit for Flow Cytometry Analysis, Invitrogen) was used to evaluate the phagocytosis capacity of ovine colostral PMN. Cells were isolated as described above and then resuspended in 400 μl of sterile PBS in the final step.

One to two × 10^4^ cells were used for each phagocytosis assay. The buffers (A and B), part of the commercial kit, were brought to RT to perform the phagocytosis assay. The cell suspension was divided into four sets of 100 μl each. Buffer B had 20 μl of pHrodo^TM^ BioParticles dissolved in it, after which it was added to preparations 3 and 4. Adequate mixing was ensured by vortexing for 30 s. Accordingly, preparations 1 and 2 served as negative controls. While mixtures 1 and 3 were placed on ice, mixtures 2 and 4 were incubated at 37°C for 15 min. After this incubation time, all preparations were placed on ice to prevent PMN phagocytosis. Lysis buffer A (100 μl) was added and, after mixing, incubated at RT for 5 min. Following this, 1 ml of buffer B was added. The mixture was incubated again at RT for 5 min. After this incubation, centrifugation was performed at 350 × g for 5 min at RT. The supernatant was carefully removed, and the cell pellet was resuspended in 0.5 ml of washing buffer provided using the phagocytosis assay kit. Since the evaluation of the samples could not be done immediately, the cells were fixed at this point. A commercial cell fixation kit (BD Cytofix/Cytoperm Fixation/Permeabilization Kit, Berlin, BD) was used for this purpose. The washing buffer was available in 10× concentration and diluted 1:10 with distilled water. The cell suspension was centrifuged at 500 × g for 5 min at RT. The supernatant was carefully poured off, and cells were resuspended in the Fix-Perm^®^ solution (Thermo Fischer Scientific). This was followed by 20 min of incubation at 4°C. After this, another centrifugation (5 min, 500 × g at RT) was performed. The supernatant was removed, and the cells were rewashed as described above. The fixed cells were resuspended in 250 μl washing buffer and analyzed at another time point using a flow cytometer (BD Accuri C6Plus) allocated at the Institute of Parasitology of the Justus Liebig University Giessen, Giessen, Germany.

### 2.8. Statistics

The total number of cells from different animals was compared by means of a *t*-test using GraphPad Prism^®^ 8 software (San Diego, CA, USA). For the evaluation of statistical significance, a level of α = 0.05 was used here. The results with *p-*values lower than or equal to 0.05 were assessed as statistically significant.

## 3. Results

### 3.1. The number of ovine colostral PMN during the first 12 h postpartum

The presence of PMN was observed in all collected ovine colostrum samples (*n* = 15). The concentration of PMN largely fluctuated among the investigated animals. Although statistically significant differences (*p* < 0.001) were observed in the number of cells between certain individual animals, no significant difference (*p* = 0.1) between the animals was observed overall. Additionally, there was no significant difference in the number of PMN between the examined complexes (*p* = 0.3) (please refer to Table 1). The average standard deviation of mean cell counts for all animals was approximately 66%. The number of PMN in the colostrum did not change significantly over the first 12 h post-partum (*p* = 0.07).

**Table 1:** Average number of PMN with standard deviation in sheep colostrum from parturition to 12 h *post-partum* (p. p.).

**Time point**	**Immediately p. p**.		**4 h p. p**.		**8 h p. p**.		**12 h p. p**.	
**Mammary complex**	**Left**	**Right**	**Left**	**Right**	**Left**	**Right**	**Left**	**Right**
PMN/ml	22,229 ± 17,008	18,467 ± 14,683	15,957 ± 8,508	15,374 ± 7,913	14,839 ± 8,372	13,110 ± 5,696	13,319 ± 8,228	15,529 ± 5,234

### 3.2. *Neospora caninum*-tachyzoites induced NETosis in ovine colostral PMN

**Figure 1:**
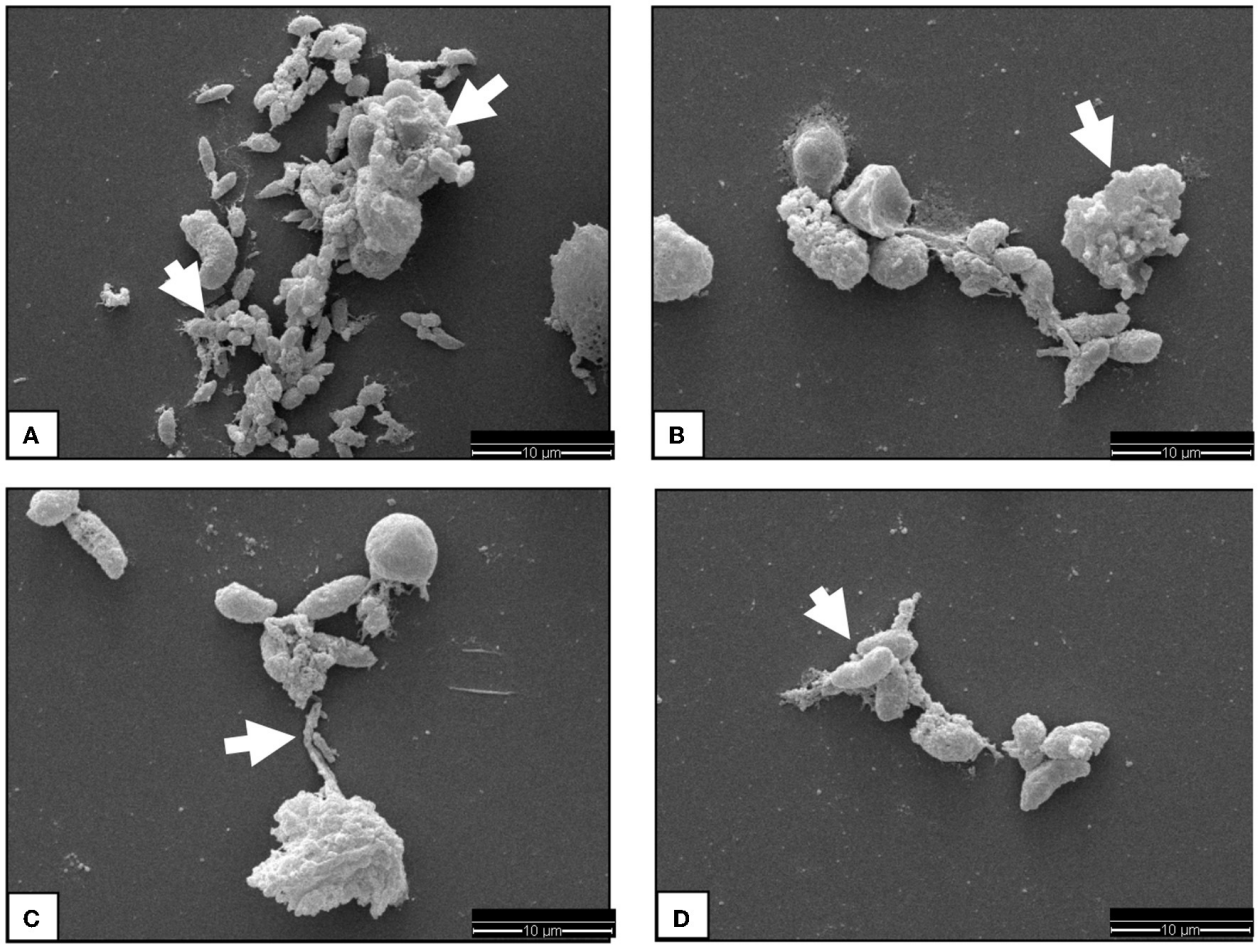
SEM analysis display of ovine colostral PMN. Image **(A)** shows some *N. caninum* tachyzoites covered by chromatin fibers just loosely on the lower part of the image, while in the upper part, a complete capture of the Tachyzoites can be observed. The cell in the upper image region has become the complete suicidal NETosis. Image **(B)** shows a chromatin fiber catching some Tachyzoites and a massively activated PMN indicated by a white arrow head. In image **(C)**, a highly activated PMN sending a chromatin fiber to already covered Tachyzoites is observable. If this is a case of vital NETosis cannot be said for sure. The image **(D)** shows some tachyzoites sticking together by chromatin fibers.

The SEM analysis was used for ultrastructural visualization of *N. caninum*-induced colostral NETosis. Suicidal NETosis was detected by SEM analysis in ovine colostrum-derived PMN exposed to live, as well as heat-inactivated (killed), *N. caninum* tachyzoites for 180 min, which triggered the formation of both thick and thin chromatin strands from dead colostral PMN (Figure 1). Additionally, SEM analysis demonstrated that not all colostral PMN responded to the stimulation as described above with NET formation. Some non-activated cells with smooth cell surfaces were observed as well. Other colostral PMNs already seemed clearly activated, which was shown by their rough and uneven surfaces. Some tachyzoites appeared to be loosely covered by excreted NET filaments, while other tachyzoites were completely enveloped within them.

**Figure 2:**
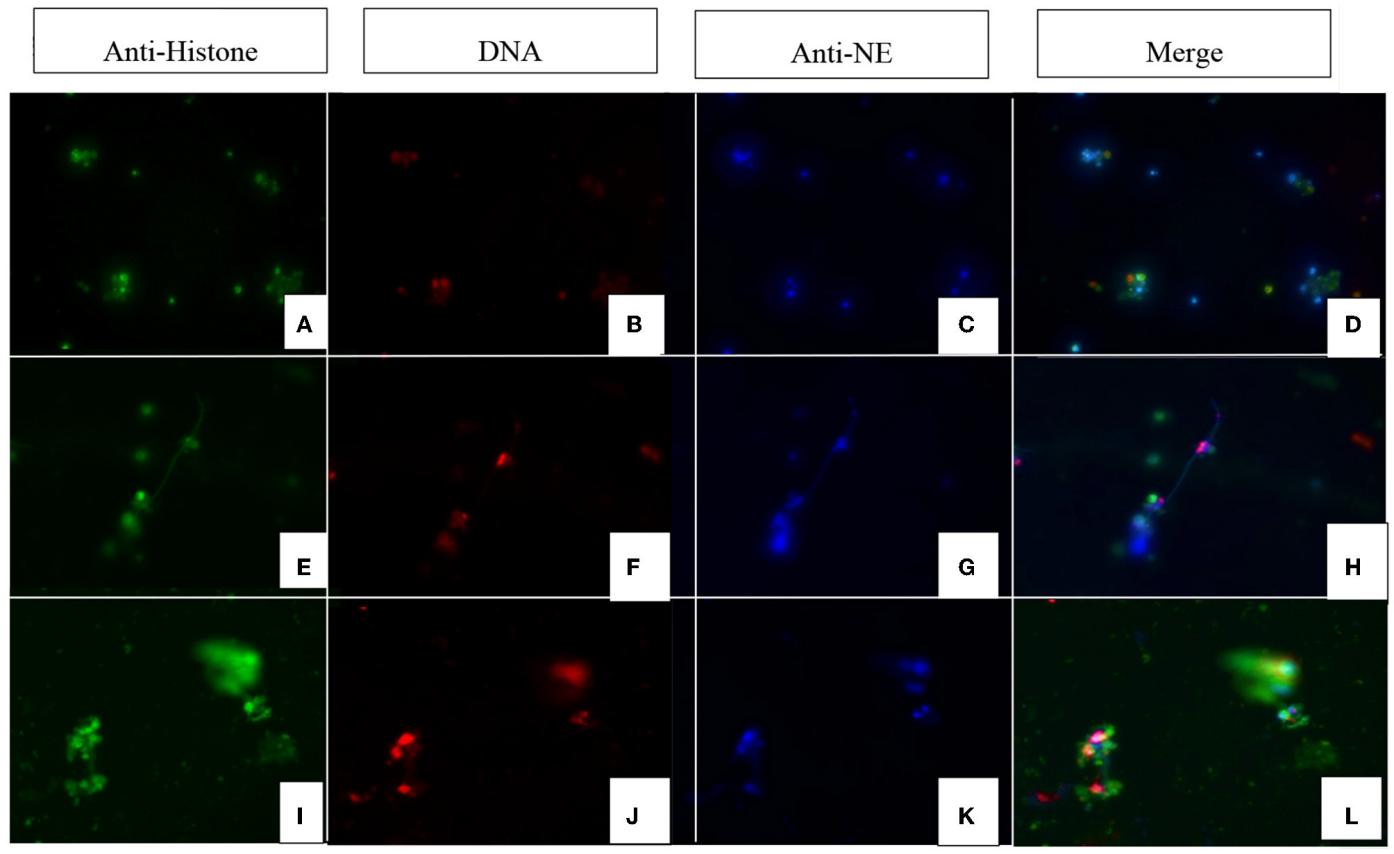
Immunofluorescence images of *Neospora caninum*-triggered ovine colostral NETs (magnification 400x). Images **(A, E, I)** show the fluorescence signal of the anti-histone antibodies; images **(B, F, J)** show the corresponding signals of the DNA dye (DAPI). Images **(C, G, K)** show the fluorescence of anti-NE (neutrophil elastase) signals and images **(D, H, L)** show the merge. Rows **(A–D)** show the control approach with unstimulated PMN. Rows **(E–H, I–L)** show PMN from different animals stimulated with vital *N. caninum* tachyzoites.

*N. caninum*-induced colostrum-derived NET formation was further visualized by immunofluorescence microscopy analysis (Figure 2). In this study, the colocalisation of PAN histones (i.e., H1, H2A/H2B, H3, and H4) and NE on effaced DNA was identified as classical NET-specific components, thereby confirming the induction of ovine colostral NETosis.

Moreover, *N. caninum*-tachyzoites triggered the formation of different NET phenotypes in exposed colostral PMN. Thus, different NET phenotypes were found in samples after incubation with *N. caninum* tachyzoites for 180 min.

None of the negative controls showed NETosis.

### 3.3. Phagocytosis test

**Figure 3:**
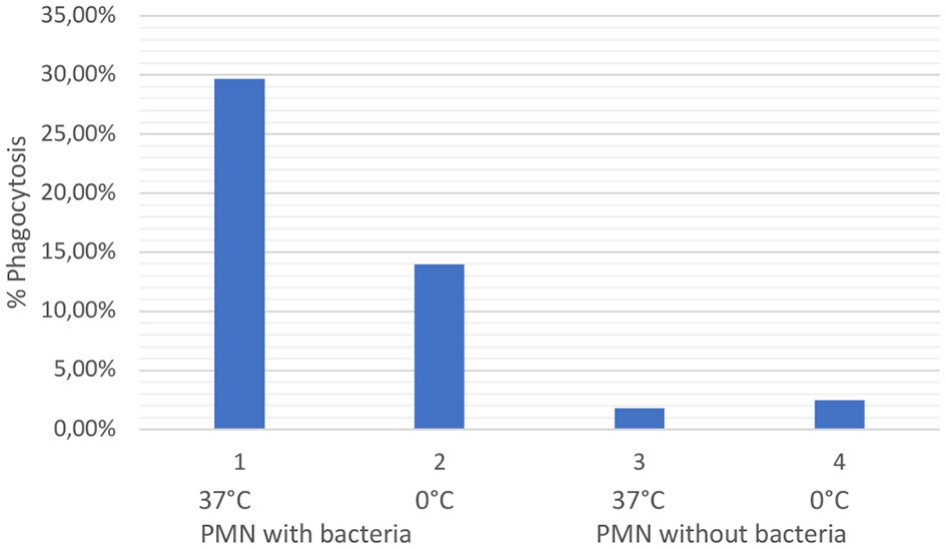
The graph shows the values of phagocytosis activity in percentages of ovine colostral PMN. The values shown in one are the values of PMN incubated with opsonized bacteria at 37°C (positive control). The values shown in two are the values illustrated for the cells with opsonized bacteria on ice. Values under three and four are the colostral PMN without bacteria at 37°C (3) and on ice (5).

Evaluation of the phagocytosis test revealed that PMN from ovine colostrum (*n* = 3) clearly demonstrated activation and phagocytosis. Thus, it can be concluded that, even after passing through the colostrum, the maternal PMN did not die but instead continued to retain its immunological properties. It is exemplary that Figure 3 illustrates the colostral PMN phagocytosis rate of one sheep. A significant difference can be observed between samples 1 and 2 compared to samples 3 and 4, which did not contain marked bacteria (positive control). Sample 1 exhibited slightly higher phagocytosis levels due to the higher reaction temperature. The reduction of phagocytosis in sample 2, with bacteria on ice, shows that the effect observed in 1 is real and not an artifact because cells phagocytise bacteria in a larger amount in warmer environments.

### 3.4. Inactivated *Neospora caninum*-tachyzoites do not hamper ovine colostral-derived NETs release

The use of heat-inactivated tachyzoites (i.e., incubation at 60°C for 10 min) yielded no changes in the NET formation reactions of exposed ovine colostral PMN. These PMN reacted to heat-inactivated (killed) tachyzoites with the formation of NET structures in the same way as they reacted to living parasites. Therefore, it can be assumed that neither intactness of the parasite nor parasite motility is a prerequisite for the formation of NETs by colostrum-derived PMN.

## 4. Discussion

The importance of colostrum for mammalian neonates, not only in sheep but also in other animal species, can hardly be overestimated. This first milk is crucial, not only with regard to the nutritive supply for the newborn but also for the transfer of maternal immunity. This immunological transfer occurs through a number of routes. A well-established transfer mechanism is through the absorption of maternal immunoglobulins (27). However, colostrum contains more than immunoglobulins (7, 28), i.e., maternal leukocytes (14, 29) to properly protect neonates after colostrum uptake. It has long since established that leukocytes of the innate immune system, including PMN contained in colostrum, transfer not only passive protection to the neonates but also perform immunological tasks thereafter in the neonate body (10, 12–14, 29). For example, Langel et al. (14) described how calves that received cell-containing colostrum had a significantly more effective immune response than calves that received cell-free, but otherwise identical, colostrum. The cell-free-fed group was also more susceptible to frequent neonatal diseases (14).

However, it has not been clear to date whether PMN in colostrum can still release NETs as an effector mechanism against invasive parasites. The present study clearly demonstrates that these cells are indeed able to fulfill immunological functions, even after incubation in the colostrum.

*N. caninum* forms tachyzoites in the intermediate host, such as those used to stimulate the cells in the present study. Thus, it can be assumed that colostral PMN and *N. caninum* tachyzoites may encounter each other in the gut (or throughout the rest of the neonate's organism) when replicating in host endothelial cells of vessels or organs. Therefore, the detection of NETosis may well be an established defense mechanism against this parasite in ovine neonates.

Vital and suicidal NETosis processes are highly efficient early innate defenses of activated PMN to immobilize and eliminate invading pathogens (21) by extruding web-like extracellular structures. These NETs consist of basic DNA structures, citrullinated global histones (H1, H2A/H2B, H3, H4), NE, and various antimicrobial peptides/proteins/enzymes, including, among others, α-defensin, lactoferrin, pentraxin, cathelicidin (LL37), cathepsin G, and calprotectin (20, 30, 31). NETosis is generally considered to be a common defense mechanism against apicomplexan parasites as well, i.e., *Eimeria bovis* (32, 33), *Toxoplasma gondii* (34–36), *Cryptosporidium parvum* (15, 37), *B. besnoiti* (38), and *N. caninum* (16). However, all these previous studies only investigated PMN isolated from peripheral animal blood, and to the best of our knowledge, the assessment of ovine colostral PMN capacities to extrude NETs against tachyzoites of *N. caninum* is unique to date.

Further evidence that PMN secreted into the colostrum are still present alive and fulfilling their usual immunological functions was demonstrated here: their NET formation capacities and their persisting phagocytosis capacity, as demonstrated by the commercial phagocytosis assays. As such, colostral PMN samples without phagocytic particles and colostral PMN samples on ice showed no phagocytic activity. This control condition highlights that the results can be attributed to true phagocytosis rather than a measurement error.

Extracting PMN from colostrum is more difficult than extracting it from peripheral blood or bone marrow, as the fat content of ovine colostrum is much higher, and PMN is relatively diminished in numbers, as already reported for canine colostrum PMN isolation (4).

The number of ovine colostral PMN did not differ much between udder sides, and instead, marked differences were observed between individual animals, as indicated by the relatively high standard deviation (66% of the observed values). Gonzalez and Santos (7) reported PMN to be 3–26% of all leukocytes present in bovine colostrum, and in line with these findings, we observed a similarly large range in the percentage of PMN in individual ovine colostrum samples.

The relatively low variation in the number of PMN in samples collected immediately after birth and 12 h after parturition differs from previous research examining immunoglobulins. These post-partum concentrations decreased at a very rapid rate and to a large extent (39). Although immunoglobulins accumulate in the udder sides over a long period of time after birth, a similar mechanism for PMN seems unlikely, as their short lifespan renders accumulation in the colostrum as rather impossible. It remains unclear whether there is an intestinal barrier that prevents the uptake of colostral leukocytes in sheep. The minor decrease in the number of PMN in the colostrum after birth may indicate that the time in which these cells influence the immune system of neonates is longer than just the initial period. While a local benefit in the intestinal lumen could be presumed, detailed analyses of these cells and their defense mechanisms in lambs after adequate colostrum uptake are required.

With regard to different *N. caninum-*triggered NET phenotypes, no clear tendency toward a certain type was found here. These phenotypes have already been described for other protozoan parasites, i.e., *Trypanosoma brucei brucei* (26) and the closely related apicomplexan parasite *Besnoitia besnoiti* by Zhou et al. (40). The interaction of *N. caninum*-tachyzoites with colostral PMN triggered mainly *agg*NETs and *diff* NETs after 3 h of co-cultivation. Some *spr*NETs were also detected, but to a much lesser extent. In colostral PMN incubated with vital *N. caninum* tachyzoites, *diff* NETs and *agg*NETs occurred in equal measure, but only *spr*NETs occurred in a significantly lesser measure in comparison to the former two. Interestingly, positive controls using Ca^++^ ionophores showed a slight tendency toward *agg*NETs (data not shown). However, these observations differ from other reports in the literature. For instance, Grob et al. (26) found that bovine PMN from peripheral blood after stimulation with vital and motile *T. b. brucei* trypomastigotes resulted primarily in *agg*NETs. Other studies with large and highly motile multicellular parasites, i.e., *Haemonchus contortus-* (25), *Dirofilaria immitis-* (25), and *Angiostrongylus vasorum*-larvae (26), demonstrated a variation depending on the size and motility of these stimulating parasites and the phenotypes of NETs formed.

To date, colostrum quality has been evaluated only in terms of immunoglobulin content (41). However, leukocytes also appear important for colostrum quality, as recently reported for canids, where colostrum-derived PMN reacted with NETosis against *N. caninum* (4). If the functions of this leukocyte population are similar to those reported previously for cattle (14), it needs further investigations, and more attention should be directed to this aspect of ovine colostrum and the implications of maternal PMN transfer on the outcome of neonatal-associated parasitosis as well as other invasive pathogens. To the best of our knowledge, this study represents the second report in the literature on colostrum-derived NETosis. Therefore, we call for further research on this neglected immunological topic, as colostral NETosis might improve neonatal protection in domestic/wild animals and humans. For this reason, it would be interesting to conduct a similar study on other animal species.

## Data availability statement

The original contributions presented in the study are included in the article/supplementary material, further inquiries can be directed to the corresponding authors.

## Ethics statement

Ethical review and approval was not required for the animal study because we used rest volumes from routine diagnostics according to the approval number kTV 8-2017. Written informed consent for participation was not obtained from the owners because we have used rest volumes from routine diagnostics.

## Author contributions

LD: did the examinations and wrote the manuscript. IC: did the evaluation of the phagocytosis test. DG: helped with laboratory work. AW: invented examination. CH, LD, and AW: edited the manuscript. IC and DG: helped with laboratory work. IC and AT: reviewed the manuscript. All authors contributed to the article and approved the submitted version.
